# Ultrasound as a New Method for the Release and Identification of Novel microRNAs and Proteins as Candidate Biomarkers in Pancreatic Cancer

**DOI:** 10.3390/cancers17121979

**Published:** 2025-06-13

**Authors:** Veronica Zelli, Alessandra Corrente, Chiara Compagnoni, Francesco Colaianni, Martina Sara Miscione, Monica Di Padova, Daria Capece, Gaetano Barbato, Edoardo Alesse, Francesca Zazzeroni, Alessandra Tessitore

**Affiliations:** 1 Department of Biotechnological and Applied Clinical Sciences, University of L’Aquila, 67100 L’Aquila, Italy; veronica.zelli@univaq.it (V.Z.); alessandra.corrente@graduate.univaq.it (A.C.); francesco.colaianni1@graduate.univaq.it (F.C.); martinasara.miscione@graduate.univaq.it (M.S.M.); monica.dipadova@univaq.it (M.D.P.); daria.capece@univaq.it (D.C.); edoardo.alesse@univaq.it (E.A.); francesca.zazzeroni@univaq.it (F.Z.); 2 Inno-Sol srl, 00165 Rome, Italy; gaetano.barbato@inno-sol.it; 3 Department of Biology, School of Pharmacy, University of Rome Tor Vergata, 00133 Rome, Italy

**Keywords:** pancreatic cancer, biomarkers, microRNA, early diagnosis, ultrasounds

## Abstract

**Background/Objectives:** Pancreatic cancer (PC) is among the most aggressive malignancies, often diagnosed at late stages. MicroRNAs (miRNAs) and proteins released from the tumor microenvironment into body fluids represent promising non-invasive biomarkers for early cancer detection. In this study, we took advantage of an innovative ultrasound (US)-based instrument (SonoWell^®^, Inno-Sol srl, Rome, Italy) to treat PC cells in order to promote and amplify the release of molecules, with the aim of identifying novel putative diagnostic PC biomarkers. **Methods:** Three human pancreatic adenocarcinoma cell lines (T3M-4, Panc02.03, and PaCa-44) and a non-cancerous pancreatic epithelial line (HPanEPic) were subjected to US using the SonoWell instrument. MiRNAs released in the supernatants were profiled by TaqMan-based qRT-PCR microfluidic cards, while proteins were analyzed by antibody arrays. Publicly available datasets of circulating miRNAs in PC patients were also reviewed. **Results:** Expression levels of 22 miRNAs in T3M-4 cells, 11 in Panc02.03, and 22 in PaCa-44, none of which were identified in the non-cancerous cell line profiling, were increased in the supernatant of US-treated as opposed to control cells. Among the statistically significant miRNAs or miRNAs common to at least two tumor cell lines, the expression levels of miR-155-5p, miR-320a, miR-32-5p, and miR-93-5p were also found to be significantly upregulated in sera from PC patients compared to the results for healthy controls. With regard to proteins released after sonication, several molecules were identified as candidate biomarkers in cancer US supernatants (Beta-2 microglobulin, CA125, CA19-9, CEA, CRP, Galectin-3, TIMP-1, uPA, and VEGF-A). **Conclusions:** We demonstrated that US-mediated sonoporation can promote and amplify the release of small molecules, miRNAs, and proteins into cell culture supernatants for consideration as putative biomarkers, thus encouraging further studies aimed at directly validating their expression levels in sera/plasma from PC patients and at deepening their role in the treatment of PC.

## Simple Summary

The identification of highly sensitive and specific biomarkers for the early diagnosis of pancreatic cancer (PC) is a fundamental goal to improve the outcome and treatment options for PC patients. In this context, microRNAs and proteins released from the tumor microenvironment into body fluids represent promising non-invasive candidates for early cancer detection. Here, we employed an innovative ultrasound (US)-based instrument to promote and amplify the release of molecules from PC cell lines in order to identify novel putative diagnostic PC biomarkers. A preliminary validation of these findings using publicly available datasets of PC patients was also performed. Overall, the results of this study highlight the suitability and utility of US-based methodologies for the identification of novel molecules to be considered as candidate non-invasive biomarkers in PC.

## 1. Introduction

Pancreatic cancer (PC) is one the most fatal malignancies, being diagnosed at the unresectable or metastatic stage in approximately 80% of patients [1]. The detection delay is attributable to the absence of specific clinical symptoms in the early stages, thus contributing to very poor 5-year and overall survival rates (5–15% and 6%, respectively in the United States). More than 90% of PCs are exocrine adenocarcinomas arising from pancreatic ductal cells, and several risk factors (e.g., smoking, alcohol consumption, diabetes, obesity, chronic pancreatitis, family history) have been described [2]. Multidetector computed tomography (MDCT), abdominal magnetic resonance imaging (MRI)/magnetic resonance cholangiopancreatography (MRCP), and to a lesser extent, ultrasonography, are used to ascertain the presence of a suspected adenocarcinoma [3]. Several minimally invasive blood tests, such as liver function tests, especially in presence of jaundice, or tumor marker levels (CA19-9, CEA), can be used to aid pancreatic cancer diagnosis, but unfortunately, these lack the necessary diagnostic specificity, sensitivity, and accuracy [4]. Overall, invasive procedures (percutaneous or endoscopic biopsy, surgery with subsequent histological analysis) remain the definitive methods to confirm diagnosis [5]. In this context, the identification of new non-invasive biomarkers to support PC diagnosis at an earlier stage could revolutionize patients’ clinical prognosis and treatment.

Therefore, the most important challenge to date is identifying novel molecules for early PC detection that can be distinguished from the physiological noise, with high specificity and sensitivity [6].

Small molecules physiologically released from the tumor microenvironment through biological membranes, such as nucleic acids (cfDNA, ctDNA, microRNA) or peptides/small proteins, are considered promising liquid biopsy-based biomarkers [6].

MicroRNAs (miRNAs) are short, non-coding RNAs able to specifically regulate the expression of target genes at the post-transcriptional level. They act by fine-tuning key physiological processes (e.g., cell proliferation, apoptosis, inflammation, metastasis), and miRNA expression dysregulation has been extensively described in several high-impact diseases (e.g., cardiovascular and neurodegenerative diseases, diabetes, cancer) [7,8,9]. MiRNAs can also be released by active or passive mechanisms from diseased tissues into the bloodstream, where they are stable and resistant to endogenous RNase [10,11,12], offering a potential source of non-invasive diagnostic, prognostic, and predictive biomarkers. Moreover, circulating miRNAs can be detected at very low concentrations in serum/plasma, thanks to the sensitive methods (e.g., quantitative PCR) available.

Ultrasound (US) and focused ultrasound (FUS) at low/moderate energy levels have been applied for diagnostic purposes due to their ability to overcome biological barriers and favor the release of small molecules, with negligible cell death, in in vitro and in vivo preclinical models [13,14,15,16]. This is attributable to the phenomenon of acoustic cavitation, generating cavities within biological membranes [17]. US waves are characterized by frequency and pressure amplitude, the latter with positive or negative values causing tissue compression or expansion, respectively. Negative pressure applied to a fluid induces cavitation bubbles (few µm in diameter) arising from gas dissolved within the medium, which can expand and shrink in size and then collapse, producing consequent mechanical stress and heating [18]. The cavitation responses associated with the rupture of biologic barriers can include five phases, with reversible or even irreversible effects in terms of cell viability, including membrane retraction, sonoporation, endo/exocytosis, membrane blebbing, and apoptosis, depending on the acoustic parameters. Membrane retraction seems to contribute to drug delivery and biomarker release due to the enlargement of cell–cell junctions [19]; sonoporation can induce the formation of pores within the lipid bilayer, mediating a bi-directional transport and leading to resealing; endocytosis and exocytosis can show protective effects against mechanical tension [20].

US-based technology has proven to be a useful tool to identify miRNAs of potential clinical utility by increasing a bidirectional flux that facilitates the release of low-molecular-weight molecules to be considered as potential biomarkers [16,21]. In this work, we used an ultrasound-based instrument for cellular research to promote and amplify the physiological process of small molecule release from PC human cell lines, thus increasing their concentration in culture supernatants and allowing for the identification of novel putative biomarkers, which were further validated on publicly available human datasets.

## 2. Materials and Methods

### 2.1. Cell Lines

Three pancreatic adenocarcinoma cell lines (T3M-4, Panc02.03, and PaCa-44) were purchased from ATCC (Manassas, VA, USA). Specifically, the T3M-4 metastatic (lymph node-derived) cells, isolated from a 64-year-old Asian male, were characterized by an epithelial-like morphology and the ability to produce the tumor marker CEA. At the molecular level, this cell line harbors the KRAS p.Gln61His and the TP53 p.Tyr220Cys oncogenic alterations. Panc02.03 and PaCa-44 are primary tumor-derived cell lines, obtained from a 70-year-old Caucasian female and a male of unspecified age, respectively. Regarding their main oncogenic alterations, Panc02.03 cells carry the KRAS p.Gly12Asp, TP53 p.Arg248Gln, CDKN2A p.Tyr44fs*1, and SMAD4 p.Arg135* variants, while the PaCa-44 cells exhibit the KRAS p.Gly12Val and TP53 p.Cys176Ser variants (https://www.cellosaurus.org/index.html and related databases, accessed on 3 June 2025).

The cells were cultured in RPMI-1640 (EuroClone, Milan, Italy) supplemented with exosome-free FBS (10%), L-glutamine (2 µm), and penicillin-streptomycin (0.05 U/mL) and were maintained at 37 °C with 5% CO_2_. As a control, a normal pancreatic epithelial cell line (HPanEpic, P10475, Innoprot, Derio, Spain) was cultured under the same conditions by using the specific EpiCM medium (Innoprot), supplemented with exosome-free FBS (2%), epithelial growth supplements (1%, Innoprot), and penicillin-streptomycin (1%, Innoprot). At 80% confluency, the cells were treated with trypsin, counted with an automatic cell counter (Cyto Smart, Corning Life Sciences, Durham, NC, USA), transferred in a Falcon 24-well plate (cod 353047, Corning Incorporated, Corning, NY, USA) (per well: 80,000 T3M-4 cells, 150,000 Panc02.03, PaCa-44, and HPanEpic cells, in 500 µL of medium) and cultured at 37 °C with 5% CO_2_ for 24 h.

### 2.2. US Treatment

After 24 h, the medium was removed, the cells were washed three times with PBS, and 300 µL of fresh serum-free medium was added to each well. Plate-wells, maintained at 37 °C, were subjected to US treatment (one single well at a time) using the SonoWell^®^ instrument (Inno-Sol srl, Rome, Italy) at 1 MHz frequency and a 460 kPa acoustic pressure (AP) negative peak. Total burst duration (TBD), duty cycle (DC), total sonication duration (SD), and thus, the corresponding acoustic energy intensity deposited (I_sppa_ and I_spta_) were adapted to each cell line: T3M-4 (TBD 6 ms, 30% DC, SD 30 min, I_sppa_ 6.995 W/cm^2^, I_spta_ 2.099 W/cm^2^); Panc02.03 and HPanEpic (TBD 2 ms, 10% DC, SD 1 h, I_sppa_ 6.995 W/cm^2^, I_spta_ 0.699 W/cm^2^); PaCa-44 (TBD 2 ms, 10% DC, SD 40 + 40 min interspersed with 10 min pause without US, I_sppa_ 6.995 W/cm^2^, I_spta_ 0.699 W/cm^2^).

Measurements of the AP were performed following suggestions of the ITRUSST consensus, where applicable, to the in-vitro measurements [22], and we also adopt their suggested nomenclature for the acoustic parameters. A manufacturer-calibrated hydrophone (Precision Acoustic, Dorchester, UK), equipped with a 0.2 mm diameter needle-type tip, detected the acoustic pressure in the far field, positioning the fully submerged well-plate on top of the 1 MHz flat transducer (12 mm diameter) at the distance of the near/far field plane, 1 mm from the well bottom. The same z-coordinate was used during subsequent experiments. The measurements determined the maximum peak of intensity by scanning the x–y plane with the hydrophone at 12 fixed z-coordinates, exploring the volume within the well. Signal detection was obtained using a Picoscope 3204A (Pico Technology, St. Neots, UK).

All sonication parameters were set up to prevent any alteration in cell morphology and to maintain over 85% cell viability. Following US treatment, cells were incubated at 37 °C and 5% CO_2_ for 10 min. Morphological alterations were evaluated by optical microscopy analysis. Cell viability was then assessed using an automatic cell counter (Cyto Smart, Corning Life Sciences) and Trypan Blue staining. The same washing, temperature, and timing conditions were applied to the untreated control samples, cultured in parallel within an incubator. Each supernatant was collected and centrifuged for 20 min at 4 °C and 1500× *g* to remove cellular debris. Samples were stored at −80 °C and used for downstream applications, as described below.

Further information on the SonoWell instrument and experimental reproducibility is available in the Supplementary Materials.

### 2.3. RNA Extraction and miRNA Expression Analysis

Total RNA, including fractions less than 200 nucleotides in length, was extracted from the US and control supernatants (300 µL) using the Plasma/Serum RNA Purification Mini kit (Norgen Biotek, Thorold, ON, Canada), following the manufacturer’s instructions. Before extraction, cel-miR-39-3p was added as exogenous control at a 5 fmol/mL final concentration. RNA quantity and quality were measured using NanoDrop 2000 spectrophotometry (Thermo Fisher, San Diego, CA, USA).

To assess the release of molecules, as a priority, we considered the expression levels of miR-23b-3p and miR-7 in the US and control supernatants from adenocarcinoma and HPanEpic cells, respectively. For this purpose, we used Thermo Fisher TaqMan small RNA assays (#245306 and #005723, respectively). RNAs were briefly retrotranscribed (TaqMan MicroRNA Reverse Transcription Kit, Applied Biosystems, Foster City, CA, USA) and analyzed (7500 Fast Real Time PCR System, Applied Biosystems) using the 2^−ΔΔCt^ method by considering the spiked-in cel-miR-39-3p as an exogenous control and comparing US vs. untreated control samples. Each experiment was repeated at least three times. Once the miRNAs’ release after sonication was ascertained, the RNAs were retrotranscribed again (TaqMan advanced miRNA cDNA synthesis kit, Applied Biosystems) and analyzed with TaqMan Advanced miRNA Human/Serum Plasma cards (Applied Biosystems) using a ViiA7 instrument (Applied Biosystems). The experiments were repeated at least three times. MiRNA expression levels were assessed using a comparative assay (2^−ΔΔCt^), and the data were normalized using the global normalization method. RT-qPCR data were analyzed by Quant Studio and Expression Suite v1.3 software (Thermo Fisher). A manual check focused on PCR amplification plot profiles was also performed, and only miRNAs with good amplification curves were retained. Differentially expressed miRNAs, showing relative quantification (RQ) ≥ 2 in the sonicated compared to the control samples, were considered for further analyses. *p*-values were calculated using the Expression Suite software using a Student’s *t*-test, and a *p*-value < 0.05 was considered statistically significant.

### 2.4. Protein Extraction and Analysis

Proteins from the US and control supernatants, obtained from single wells as previously described, were extracted using phosphatase inhibitor (0.1 mM PMSF, 1 µg/mL aprotinin, 10 mM NaF, 0.1 M Na3VO4) and a Complete-Mini protease inhibitor cocktail tablet (Roche Molecular Biochemicals, Basel, Switzerland) and centrifuged at 1500× *g* for 20 min at 4 °C. The protein concentration was quantified using the Pierce BCA protein assay kit (Thermo Fisher) at 562 nm, according to the manufacturer’s instructions. Protein expression levels were analyzed using the Human Cancer Discovery Array C3 (RayBiotech, Peachtree Corners, GA, USA), based on the manufacturer’s instructions, allowing us to evaluate the expression profile of 30 proteins involved in cancer biology. Chemiluminescent signals were collected using ImageQuant LAS 4000 (GE Healthcare, Tokyo, Japan), and densitometric analysis was performed using ImageJ software v 1.53e, based on the RayBiotech algorithm.

Proteins with a fold increase ≥ 1.3 (signal increase ≥ 30%) in US-treated tumor cells compared to that of the controls were considered upregulated. The analyses were performed in duplicate, and only results confirmed in both experiments were considered.

### 2.5. Publicly Available Datasets for Results Validation

Four publicly available datasets, EXP00529 (GSE106817) [23], EXP00620 (GSE112264) [24], EXP00609 (GSE113740) [25], and EXP00538 (GSE113486) [26], from the Database of Differentially Expressed miRNAs in Human Cancers (dbDEMC, https://www.biosino.org/dbDEMC/index, accessed on 20 January 2025), were used to validate the expression levels of miRNAs released in the US supernatants in the PC patients and healthy controls (Supplementary Table S1).

Notably, in these studies, miRNA profiling was performed on serum samples from cancer patients and healthy controls. Data were filtered based on a log2 fold change (FC) ≥ 0.58 and an adjusted *p*-value (padj) ≤ 0.05.

GSE106817 and GSE112264 datasets were also used to perform receiver operating characteristic (ROC) curve analysis in order to estimate the miRNAs’ diagnostic value (individually or in combination) in discriminating PC cases from non-cancer samples, as well as from other cancer types, using the CombiROC R package [27]. The analysis was performed using R software v 4.3.1 (www.r-project.org).

### 2.6. Network and Pathway-Based Analysis

The multiple query module of the miRNet 2.0 tool (https://www.mirnet.ca/, accessed on 3 June 2025), which allows for the loading different input types (in this case, miRNAs and proteins), was used to identify the most relevant connections among the biomarkers emerging in this study. The STRING v10 protein–protein interaction (PPI) database was also considered to obtain a more comprehensive overview of the biological context of the identified miRNAs and proteins. Starting from the first-order network, a minimal subnetwork that maximally connects the seed nodes was extracted, thereby simplifying and focusing the network analysis. MiRNA enrichment analysis was performed using the explorer function of the miRNet tool, while the pathway analysis of all proteins in the network was conducted using the Database for Annotation, Visualization, and Integrated Discovery (DAVID) (https://davidbioinformatics.nih.gov/; accessed on 3 June 2025), based on the Kyoto Encyclopedia of Genes and Genomes (KEGG) and Reactome Pathway databases. An adjusted *p*-value ≤ 0.05 was applied to filter statistically significant terms.

## 3. Results

### 3.1. Assessment of Effective Molecule Release After US Treatment

**Figure 1:**
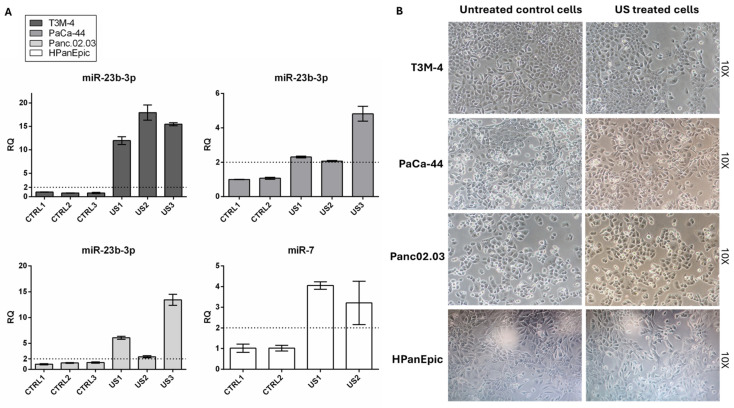
US parameter settings and validation. (**A**) miR-23b-3p and miR-7 expression levels in US-treated (US) compared to control (CTRL) supernatants from adenocarcinoma (T3M-4, PaCa-44, and Panc02.03) and HPanEpic cells, respectively, confirming US-mediated miRNA release (RQ ≥ 2, *p* < 0.05); each experiment was performed in triplicate under the same conditions. (**B**) US treatment parameters were set up to produce no morphological changes and more than 85% viable cells. Abbreviations: relative quantification (RQ).

Due to both the biological and technical variability in the cell response to US treatment, related to factors such as cell type, cell cycle phase, ultrasound parameters, and environmental culture conditions [16,28], the efficacy of US treatment was verified by evaluating the expression levels of miR-23b-3p and miR-7, which are known to be expressed in both PC and normal pancreatic cells, respectively [29,30,31]. The expression levels of these two miRNAs were evaluated in the US-treated and control supernatants from adenocarcinoma and HPanEpic cells, respectively, and only samples in which US-mediated release was confirmed (RQ ≥ 2) were considered for miRNA/protein profiling analysis (Figure 1A).

Optical microscopy showed that the US parameters employed did not induce any significative alterations in cell morphology or adhesion properties (Figure 1B). Moreover, automatic cell count and Trypan Blue staining revealed more than 85% viable cells, demonstrating that US promoted miRNA release, without significant changes in cell morphology or viability, thus confirming the suitability of the experimental conditions.

### 3.2. MiRNA Profiling in US Supernatants

**Figure 2:**
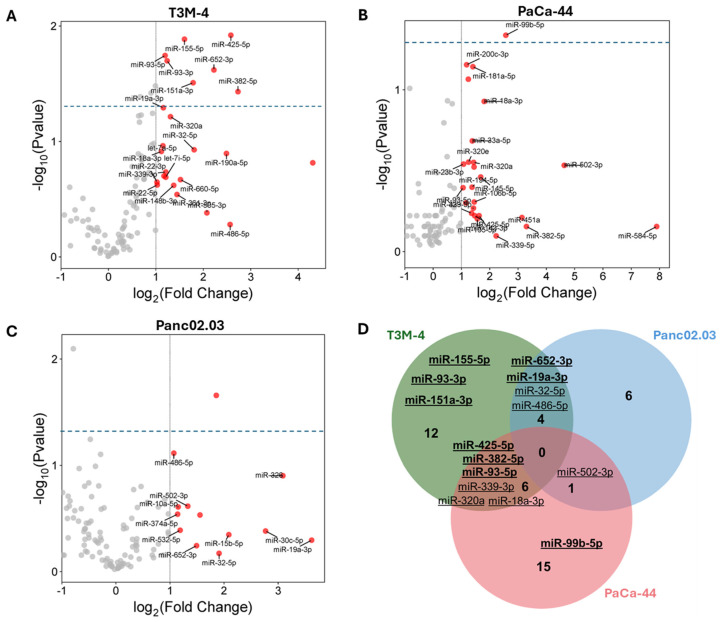
Profiling of PC US-released miRNAs. Volcano plots of miRNAs released from T3M-4 (**A**), PaCa-44 (**B**) and Panc02.03 (**C**) cells after sonication; only miRNAs identified in cancer cell lines but not in non-cancerous HPanEpic cells are labeled. (**D**) Venn diagram showing the eight and one statistically significant miRNAs in T3M-4 and PaCa-44 cells, respectively (highlighted in bold), as well as non-significant upregulated miRNAs (RQ ≥ 2) common to at least two tumor cell lines (n = 6), for a total of 15 miRNAs considered for further analysis.

In the US supernatants of T3M-4 and PaCa-44 cells, eight (miR-19a-3p, -151a-3p, -382-5p, -93-3p, -93-5p, -652-3p, -155-5p, -425-5p) and one (miR-99b-5p) significantly differentially expressed miRNAs were identified, respectively. None of these miRNAs were detected in supernatants from normal HPanEpic cells. In addition, we extended the analysis to the released non-significant miRNAs: in this case, 22 miRNAs in T3M-4 cells, 11 miRNAs in Panc02.03, and 22 miRNAs in PaCa-44, none of which were identified in supernatants from the non-cancerous cell line, were found to be overexpressed after US (Figure 2A–C, Supplementary Table S2). Among them, we further focused on the analyses of 15 miRNAs that were statistically significant (miR-19a-3p, -151a-3p, -382-5p, -93-3p, -93-5p, -652-3p, -155-5p, -425-5p and -99b-5p) or non-significant, but common to at least two tumor cell lines (miR-32-3p, -486-5p, -339-3p, -320a, -18a-3p, and -502-3p) (Figure 2D).

### 3.3. In Silico Validation of Differentially Expressed miRNAs

In order to preliminarily validate miRNAs as putative PC biomarkers, an in silico analysis of publicly available datasets obtained from the Database of Differentially Expressed miRNAs in Human Cancers (dbDEMC) was performed (Table 1). According to our results, significant upregulation of miR-151a-3p, -155-5p, -320a, -32-5p, -339-3p, -652-3p, -93-3p, -93-5p, and -502-3p was observed in sera from PC patients. In particular, miR-155-5p, miR-320a, miR-32-5p, and miR-93-5p emerged as the most interesting, being differentially expressed in at least two out of four datasets investigated (Table 1).

**Figure 3:**
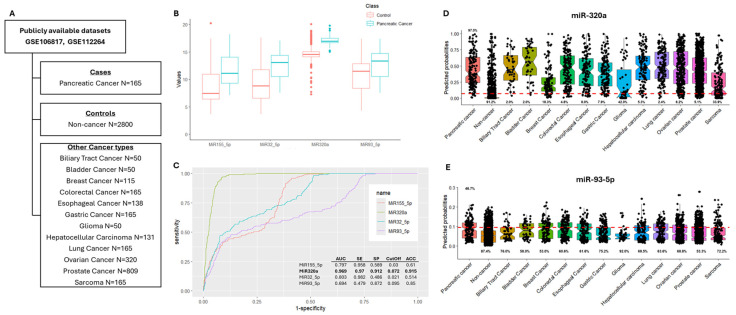
Evaluation of diagnostic potential of miR-155-5p, miR-320a, miR-32-5p, and miR-93-5p. (**A**) Study series analyzed. (**B**) Boxplots of the distribution of each miRNA in pancreatic cancer and non-cancer controls; the corresponding ROC curves and performance parameters are reported in (**C**). The ability of miR-320a (**D**) and miR-93-5p (**E**) to correctly distinguish PC patients, not only from non-cancer controls but also from the other cancer types, is displayed. The red dotted line indicates the optimal cutoff based on ROC curve analysis for PC cases and non-cancer controls. Abbreviations: area under the curve (AUC); sensitivity (SE); specificity (SP); accuracy (ACC); receiver operating characteristic (ROC).

The CombiROC R package was used to estimate the diagnostic value of miR-155-5p, miR-320a, miR-32-5p, and miR-93-5p in discriminating PC from non-cancer samples, as well as from other cancer types, using the two publicly available datasets GSE106817 and GSE112264 (Figure 3A). Notably, the two datasets include different samples (female and male patients, respectively) from the same study series, analyzed using the same method [23,24]. Therefore, by using the CombiROC package separately with each dataset or with the merged dataset, we obtained similar results.

Based on ROC curve analysis, miR-320a showed the highest diagnostic potential, with an AUC value of 0.969, a sensitivity of 0.97, a specificity of 0.912, and accuracy of 0.915, highlighting the ability of this miRNA to distinguish PC cases from healthy controls, with good efficiency (Figure 3B,C).

However, using the predicted probability function to classify the other cancer types according to the optimal cutoff obtained from the previously described ROC curves (PC cases vs. healthy controls), the results revealed the low specificity of the model in discriminating PC cases from other cancers (Figure 3D).

Similar results were observed using different miRNA combinations (Supplementary Table S3, Supplementary Figure S1)

On the other hand, although miR-93-5p showed moderate diagnostic potential in discriminating PC cases from non-cancer controls (AUC 0.694, sensitivity 0.479, specificity 0.872, and accuracy 0.85), mainly due to low sensitivity (Figure 3C), this miRNA performed better than miR-320a in terms of specificity when other tumor types were considered (Figure 3E).

### 3.4. US Protein Secretome Analysis

**Figure 4:**
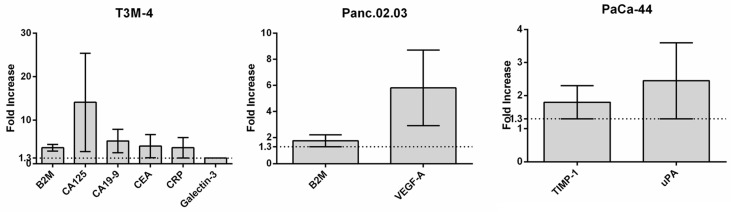
Profiling of PC US-released proteins. Bar plots showing proteins released from the three tumor cell lines after sonication compared to the results for the control cells.

To evaluate protein factors to be considered as putative PC biomarkers, the release of which was promoted and amplified by US, proteins were also extracted from supernatants and analyzed by antibody arrays. A total of nine factors were found to be more highly expressed in US-treated compared to non-treated cell supernatants, none of which were observed in non-cancerous cell line profiling. These include well-known tumor markers (e.g., CA19-9, CEA, and CA125), as well as proteins more recently described as potential novel biomarkers for the detection of PC (e.g., Beta-2 microglobulin (B2M), TIMP-1, CRP, Galectin-3, uPA, and VEGF-A) (Figure 4).

### 3.5. Analysis of the Biological Role and Molecular Interactions of the Identified Biomarkers

To determine the potential molecular interactions and biological relevance of the identified miRNAs and proteins, a network was created and pathway-based analysis was performed, as described in the methods section.

**Figure 5:**
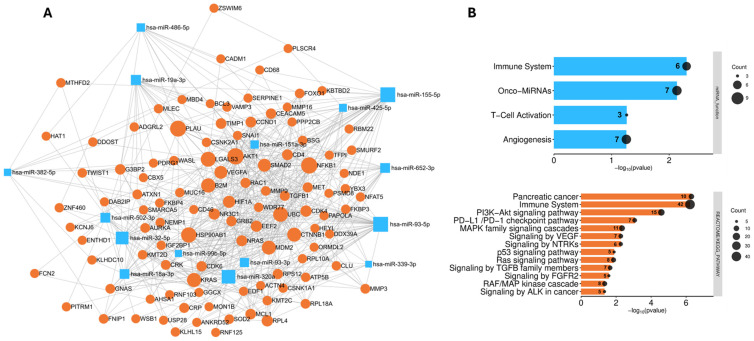
Network and pathway-based analysis of miRNAs (n = 15) and proteins (n = 9) identified in the present study, further enriched using a STRING v10 protein–protein interaction (PPI) database. MiRNA–protein and protein–protein interactions were derived from a minimal sub-network that maximally connects the different seed nodes (**A**), and related enrichment analysis was performed separately for miRNAs (n = 15) and all proteins (n = 107). Only the terms considered as the most representative and interesting among the significant expressions are reported. The miRNA and protein counts for each pathway are shown within the bars (**B**).

Overall, a network comprising a total of 122 nodes (miRNAs/proteins) was obtained (Figure 5A). Pathway analysis revealed overlapping biological processes involving both miRNAs and proteins, including immune regulation and angiogenesis. Protein enrichment analysis also highlighted pathways of interest known to be involved in PC development and progression, such as the Ras signaling pathway and the RAF/MAP kinase cascade, the p53 signaling pathway, and signaling by TGFB family members (Figure 5B).

## 4. Discussion

PC is one of the most aggressive type of tumors, typically characterized by late-stage diagnosis and poor prognosis due in part to the lack of early symptoms and the absence of routine screening programs [1]. This highlights the urgent need to identify new biomarkers, characterized by high sensitivity and specificity, to improve the outcome and clinical management of PC patients. In this study, using SonoWell^®^, an innovative US-based instrument designed for cellular studies on well-plates to promote and amplify the release of small molecules in PC cell lines, we identified novel putative miRNAs/proteins as non-invasive biomarkers for the early detection of PC.

The different cellular effects triggered by US, ranging from sonoporation/endo-exocytosis to apoptosis/necrosis, are highly dependent on the physical and biophysical parameters of the US applied [32]. Exploiting US waves to transiently permeabilize cell membranes can facilitate the uptake of molecules while maintaining cell viability, and the use of US as a new therapeutic tool in cancer has garnered great attention in recent years due to its potential to improve drug delivery [32]. Sonication can also be effective in amplifying the release of small intracellular molecules in body fluids, suggesting that this technique could be considered useful for earlier detection of cancer lesions, for example, in the presence of precancerous lesions or for the identification of prognostic biomarkers [14,33]. In this scenario, the on-site application of US waves in cancer patients, with the following quantification of released biomarkers, could represent an additional tool for a more precise diagnosis/prognosis, for which additional specific preclinical and clinical studies are required. However, it is important to emphasize that using an US-based approach in this context would require the application of FUS with low power, without any ablation, to a precise target area of the body characterized by a known lesion and only to superficial organs accessible to FUS; these are just some of the current limitations to its use in a clinical setting. In this study, we proposed the use of US as a research method to guide the identification of potential biomarkers suitable for supporting PC diagnosis. Therefore, the translational applicability is mainly related to the analysis of such molecules, identified in the in vitro models, in serum/plasma from cancer patients in order to confirm their expression level modulation and diagnostic potential.

Among the miRNAs identified here and validated in external publicly available datasets, miR-155-5p, -320a, -32-5p, and 93-5p emerged as the most interesting putative biomarkers for PC diagnosis.

MiR-155, in both its -5p and -3p forms, is considered an oncogenic miRNA widely studied for its role in promoting cell survival, proliferation, migration, and invasiveness [34,35]. A recent meta-analysis showed that tissue miR-155 is a valuable diagnostic and prognostic biomarker in most solid tumors, including PC [36]. Moreover, several studies observed the upregulation of miR-155-5p in the plasma/serum of PC patients compared to levels in healthy controls, also highlighting the possible assessment of this miRNA, alone or in combination with other miRNAs/proteins, as a biomarker for early PC diagnosis [37,38,39,40].

In contrast, miR-32-5p was predominantly described as a tumor suppressor in different cancer types [41,42,43], and few data are currently available regarding its expression levels and putative role as a circulating biomarker in cancer.

MiRNA-93-5p is involved in gemcitabine resistance in PC through targeting the PTEN-mediated PI3K/Akt signaling pathway [44]. Circulating levels of miR-93-5p emerged as relevant indicators in various cancer types, particularly PC and colorectal cancers, where high miR-93-5p expression has been correlated with disease and progression, suggesting its possible consideration as a diagnostic and prognostic biomarker [45,46]. However, the diagnostic value of miR-93-5p was found to be quite moderate, being able to discriminate PC patients from healthy controls with a sensitivity and specificity of 60% and 77%, respectively [45].

Conflicting data are available regarding the role of miR-320a in human cancers, suggesting that the oncogenic or suppressive function of this miRNA is context-dependent [47]. MiR-320a was mainly downregulated and described as a tumor suppressor in cancers such as colorectal, breast, lung, and gastric cancer [48,49,50,51,52,53]. However, in malignant pleural mesothelioma, Costa et al. reported that the upregulation of miR-320a in cell lines promotes cell proliferation, viability, and migration, whereas its stable silencing resulted in contradictory effects [47]. Similarly, Yao et al. found that miR-320a/c/d ectopic expression increased the migration and invasion of hepatocarcinoma cells [54]. Wang et al. showed that the upregulation of miR-320a promotes the proliferation, migration, and invasion of PC cells, thereby reducing 5-FU sensitivity [55].

Interestingly, in analyzing the expression levels of a panel of 17 circulating miRNAs, miR-320a and miR-93-5p emerged among the miRNAs with the highest ability to discriminate PC patients and individuals with preneoplastic lesions from healthy controls, with AUC results of 0.85 and 0.80, respectively. Furthermore, using the combination of miR-33a-3p+miR-320a+CA19.9, an AUC value of 0.95, a sensitivity of 93%, and a specificity of 85% were achieved, suggesting that this combination could represent a significant biomarker signature for the non-invasive early diagnosis of PC [56].

MiR-320a and miR-93-5p are the two miRNAs that emerged as the most promising in our study: miR-320a showed high diagnostic potential to discriminate PC cases from healthy controls (AUC of 0.969, sensitivity of 0.97, specificity of 0.912, and accuracy of 0.915), but low specificity for PC compared to other tumor cases. On the other hand, miR-93-5p showed worse performance parameters, in particular, lower sensitivity, in distinguishing PC cases from non-cancerous controls (AUC of 0.694, sensitivity of 0.479, specificity of 0.872, and accuracy of 0.85), but higher specificity for a subset of PC (~47%) compared to other cancer types.

The differences in circulating biomarkers based on molecular cancer subtypes are significant, particularly in breast cancer, where distinct profiles can inform about diagnosis, prognosis, and treatment strategies. Studies indicate that circulating biomarkers, including proteins, metabolites, and microRNAs, can vary across molecular subtypes, reflecting the underlying biological heterogeneity of cancers [57,58]. Although PC subtypes do not currently guide the clinical management of patients, increasing evidence based on omics analysis is currently defining PC subgroups with distinct biology and potential subtype-specific therapeutic vulnerabilities, offering the opportunity to define new possible clinically applicable molecular classification [59]. Therefore, it can be hypothesized that the complexity of cancer biology, including that of PC, underscores the need for further investigation into the implications and utility of circulating biomarkers, considering factors such as molecular subtypes. Notably, to date, no data is available regarding specific biomarkers associated with PC molecular subtypes. However, considering the only partially overlapping (common) miRNAs identified across the three cell lines, we can hypothesize that differences observed may be in part explained by the distinct molecular characteristics of each cell line.

We also analyzed protein factors released in the supernatants of US-treated cancer cells, detecting well-known tumor markers (e.g., CA19-9, CEA, and CA125) [4], as well as proteins as candidate novel biomarkers for the detection of PC, particularly B2M, CRP, TIMP1, and Galectin-3 [60].

CA19-9 is currently the most widely used non-invasive biomarker for PC diagnosis, and although several studies have recommended the combination of CA19-9, CA-125, and CEA as a routine screening method for PC detection, these factors are ineffective as early markers due to their low specificity [60].

Among the novel suggested protein biomarkers, B2M (in this study, common to two PC cell lines) and CRP were recently included in a PC diagnostic panel comprising 11 candidate factors which demonstrated significant performance in terms of specificity and sensitivity in discriminating PC from control samples [61]; CRP has also been suggested as a PC prognostic factor in combination with CA19-9 [62].

Similarly, TIMP-1 has been reported as a potential biomarker for the early detection of PC [60], particularly in familial PC in association with LCN2 [63], while Galectin-3 was described as a putative biomarker for screening and diagnosis, as well as an independent prognostic indicator in PC [64].

In the supernatants of US-treated cancer cells, we also detected upregulated levels of uPA and VEGF-A, the first of which has been reported to be positively associated with increased PC severity and poor clinical outcome [65], while the latter is generally found to be significantly elevated in cancer patients, including those with PC, compared to the results for healthy individuals [66].

Interestingly, the network and pathway analysis of identified miRNAs and proteins highlighted their involvement in key biological processes related to PC development and progression, which are closely associated with oncogenic driver alterations that mainly affect the *KRAS*, *TP53*, *CDKN2A*, and *SMAD4* genes [67]. Indeed, among the most representative and relevant pathways, the Ras signaling pathway and the RAF/MAP kinase cascade, the p53 signaling pathway, and signaling by TGFB family members emerged, in addition to the modulation of VEGF signaling/angiogenesis and the modulation of the immune system. These findings suggest that molecules identified in this study may function not only as diagnostic biomarkers, but also as active contributors to PC tumorigenesis. However, further investigations are required to elucidate their role in PC pathogenesis.

Overall, due to the complexity of tumor biology and the heterogeneity of cancers, our results, in agreement with data reported in the literature, show that the most successful strategy for the identification of biomarkers of clinical utility could be a combination of factors (e.g., miRNA/proteins), also taking into account the possible pathological and/or molecular features of tumors.

Studies have shown that biomarker panels outperform single biomarkers [68,69], suggesting that the factors identified here could be part of such effective combinations.

This leads to the main limitations of the present study, which, in addition to the imbalance in the number of cases and controls in the publicly available datasets queried, relate to the inability to directly analyze our own PC study series to test the diagnostic potential of the different combinations of miRNA/protein described.

## 5. Conclusions

In conclusion, we demonstrated the suitability of US-mediated sonoporation to promote and amplify the release of miRNAs and proteins in PC cell lines, highlighting the use of this method to identify candidate circulating biomarkers. This study introduces novel molecules to be considered as non-invasive biomarkers in PC (e.g., miR-320a, miR-93-5p, B2M, CRP, and TIMP-1), encouraging further studies, including those of a prospective nature, aimed at deepening the understanding of their role in PC and to support the validation of different miRNA/protein combinations directly in the sera/plasma of PC patients for early diagnostic and prognostic purposes. Advances in high-throughput sequencing, the development of standardized protocols, and analysis of large well-characterized series are essential to improve the identification of non-invasive molecular biomarkers of clinical relevance.

## Figures and Tables

**Table 1:** Studies from dbDEMC (https://www.biosino.org/dbDEMC/index, (accessed on 20 January 2025)) reporting statistically significant differences (log2FC ≥ 0.58; adj *p*-value < 0.05) in serum of PC samples compared to normal samples for the 15 selected miRNAs; among them, miR-155-5p, miR-320a, miR-32-5p, and miR-93-5p emerged as the most interesting, as they were found to be deregulated in more than one dataset.

				EXP00529 (GSE106817)		EXP00620 (GSE112264)		EXP00609 (GSE113740)		EXP00538 (GSE113486)	
miRNA	T3M-4	Pnc02.03	PaCa-44	log2FC	adj *p*-Value	log2FC	adj *p*-Value	log2FC	adj *p*-Value	log2FC	adj *p*-Value
hsa-miR-151a-3p	0.031					1.466	0.005				
hsa-miR-155-5p	0.013			1.502	1.15 × 10^−13^	1.337	0.0006				
hsa-miR-18a-3p											
hsa-miR-19a-3p	0.050										
hsa-miR-320a				1.907	5.62 × 10^−129^	1.795	5.808 × 10^−23^	1.572	2.93 × 10^−15^	1.643	4.848 × 10^−21^
hsa-miR-32-5p				1.719	2.932 × 10^−14^	2.420	9.082 × 10^−7^			1.358	0.003
hsa-miR-339-3p				1.014	6.419 × 10^−8^						
hsa-miR-382-5p	0.037										
hsa-miR-425-5p	0.012										
hsa-miR-486-5p											
hsa-miR-652-3p	0.024			0.959	1.773 × 10^−7^						
hsa-miR-93-3p	0.020			0.977	1.12 × 10^−5^						
hsa-miR-93-5p	0.018			0.721	0.003	1.527	0.001				
hsa-miR-502-3p				0.583	0.002						
hsa-miR-99b-5p			0.046								

## Data Availability

The original contributions presented in the study are included in the article. Further inquiries can be directed to the corresponding author. Publicly available datasets analyzed in this study are available in the dbDEMC database (https://www.biosino.org/dbDEMC/index (accessed on 20 January 2025)) and GEO repository (https://www.ncbi.nlm.nih.gov/geo/query/acc.cgi?acc=GSE106817, (accessed on 20 January 2025), https://www.ncbi.nlm.nih.gov/geo/query/acc.cgi?acc=GSE112264 (accessed on 20 January 2025)).

## Supplementary Materials

The following supporting information can be downloaded at: https://www.mdpi.com/article/10.3390/cancers17121979/s1, Table S1: Publicly available datasets; Table S2: List of released miRNAs identified in PC cell line; Table S3: Evaluation of the diagnostic potential of the most interesting emerging miRNAs; Figure S1: Overview of the diagnostic potential of the best performing miRNA combinations, along with additional information on SonoWell instrument specifications and experimental reproducibility.

## Abbreviations

The following abbreviations are used in this manuscript:

ACC	Accuracy
AP	Acoustic Pressure (negative peak)
AUC	Area Under the Curve
B2M	Beta-2 microglobulin
dbDEMC	Database of Differentially Expressed miRNAs in Human Cancers
DC	Duty Cycle
FC	Fold Change
FUS	Focused Ultrasound
I_sppa_	Intensity of Spatial Peak Pressure Average
I_spta_	Intensity of Spatial Peak Time Average
miRNA	microRNA
PC	Pancreatic Cancer
ROC	Receiver Operating Characteristic
RQ	Relative Quantification
SE	Sensitivity
SP	Specificity
TBD	Tone Burst Duration
US	Ultrasound
