# Supplementary material for: Ultrasound as a New Method for the Release and Identification of Novel microRNAs and Proteins as Candidate Biomarkers in Pancreatic Cancer
Source: Cancers (Basel). 2025 Jun 13;17(12):1979. doi: 10.3390/cancers17121979 (PMC12191226; doi:10.3390/cancers17121979)
Supplement: Supplementary file 1 [file cancers-17-01979-s001.zip › cancers-3656023-supplementary.pdf]

Supplementary Table S1. Publicly available datasets. Studies analyzed for circulating miRNAs expression levels in pancreatic cancer (PC) *vs* normal blood from DEMC database (https://www.biosino.org/dbDEMC/index)

Dataset blood (PC vs Normal)					
**Experiment ID**	**GEO ID**	**N cases**	**N controls**	**Technology**	**Reference**
EXP00529	GSE106817	115	2759	Microarray	Yokoi, Akira et al. "Integrated extracellular microRNA profiling for ovarian cancer screening." Nature communications vol. 9,1 4319. 17 Oct. 2018, doi:10.1038/s41467-018-06434-4
EXP00620	GSE112264	50	41	Microarray	Urabe, Fumihiko et al. "Large-scale Circulating microRNA Profiling for the Liquid Biopsy of Prostate Cancer." Clinical cancer research : an official journal of the American Association for Cancer Research vol. 25,10 (2019): 3016-3025. doi:10.1158/1078-0432.CCR-18-2849
EXP00609	GSE113740	25	969	Microarray	Yamamoto, Yusuke et al. "Highly Sensitive Circulating MicroRNA Panel for Accurate Detection of Hepatocellular Carcinoma in Patients With Liver Disease." Hepatology communications vol. 4,2 284-297. 19 Dec. 2019, doi:10.1002/hep4.1451
EXP00538	GSE113486	40	100	Microarray	Usuba, Wataru et al. "Circulating miRNA panels for specific and early detection in bladder cancer." Cancer science vol. 110,1 (2019): 408-419. doi:10.1111/cas.13856

Supplementary Table S2. List of released miRNAs identified in PC cell lines. Full list of the 44 unique miRNAs released (RQ≥2 in grey) in at least one tumor cell line, none of which identified in non-cancerous cell line profiling. Statistically significant p-values are in bold.

	**T3M-4**		**Panc02.03**		**PaCa-44**	
	**RQ**	**p-value**	**RQ**	**p-value**	**RQ**	**p-value**
let-7a-5p	2.216	0.109	1.593	0.700	1.511	0.391
let-7i-5p	2.312	0.204	na	na	1.089	0.866
miR-148b-3p	2.596	0.240	0.932	0.858	0.987	0.700
miR-151a-3p	3.447	**0.031**	0.952	0.888	1.133	0.877
miR-155-5p	3.037	**0.013**	0.515	0.700	2.229	0.500
miR-18a-3p	2.167	0.122	na	na	3.491	0.118
miR-190a-5p	5.609	0.127	1.298	0.533	0.527	0.800
miR-19a-3p	2.231	**0.050**	12.297	0.506	1.152	0.781
miR-22-3p	2.033	0.225	0.726	0.335	0.710	0.432
miR-22-5p	2.041	0.238	0.740	0.723	na	na
miR-320a	2.476	0.061	0.720	0.057	2.699	0.281
miR-32-5p	3.500	0.118	3.753	0.673	na	na
miR-339-3p	2.311	0.184	0.580	0.312	2.562	0.579
miR-361-3p	2.725	0.288	1.620	0.740	0.892	0.700
miR-382-5p	6.639	**0.037**	0.839	0.779	9.830	0.700
miR-425-5p	5.982	**0.012**	1.380	0.158	2.768	0.600
miR-486-5p	5.916	0.524	2.101	0.077	na	na
miR-505-3p	4.215	0.415	1.623	0.621	na	na
miR-652-3p	4.673	**0.024**	2.811	0.570	na	na
miR-660-5p	2.865	0.214	na	na	na	na
miR-93-3p	2.358	**0.020**	0.506	0.700	na	na
miR-93-5p	2.288	**0.018**	0.859	0.251	2.080	0.403
miR-10a-5p	0.886	0.925	2.220	0.246	1.674	0.406
miR-15b-5p	0.895	0.854	4.252	0.449	1.576	0.746
miR-30c-5p	1.499	0.301	6.813	0.415	na	na
miR-326	na	na	8.504	0.125	na	na
miR-374a-5p	1.127	0.897	2.206	0.288	na	na
miR-502-3p	na	na	2.512	0.242	24.801	0.293
miR-532-5p	0.777	0.565	2.278	0.408	na	na
miR-106b-5p	1.804	0.186	0.579	0.008	2.734	0.494
miR-143-3p	na	na	na	na	3.085	0.600
miR-145-5p	na	na	0.691	0.654	3.206	0.346
miR-181a-5p	1.679	0.212	1.143	0.389	2.648	0.072
miR-194-5p	1.320	0.819	na	na	2.596	0.400
miR-195-5p	1.374	0.335	1.958	0.286	2.954	0.627
miR-200c-3p	1.578	0.275	na	na	2.272	0.070
miR-23b-3p	1.941	0.104	1.829	0.700	2.106	0.288
miR-320e	1.551	0.167	1.079	0.886	2.381	0.280
miR-339-5p	0.628	0.465	1.308	0.572	4.685	0.800
miR-33a-5p	1.081	0.847	0.676	0.394	2.608	0.207
miR-423-5p	1.876	0.107	0.706	0.349	2.685	0.539
miR-451a	na	na	na	na	8.802	0.616
miR-584-5p	1.223	0.759	na	na	238.287	0.700
miR-99b-5p	0.751	0.557	0.672	0.497	5.933	**0.046**

Supplementary Table S3. Evaluation of the diagnostic potential of the most interesting miRNAs emerged. Results of the Receiver-Operating Characteristic (ROC) curves analysis of miR-155-5p, miR-320a, miR-32-5p, miR-93-5p and all possible combinations, to discriminate pancreatic cancer patients from non-cancer controls. The most promising single miRNAs/combinations are highlighted in bold. Abbreviations: Area Under the Curve (AUC), sensitivity (SE), specifcity (SP), accuracy (ACC), true negative (TN), true positive (TP), false negative (FN), false positive (FP), negative and positive predictive values (NPV and PPV respectively). Of note, the low PPVs are mainly attributable to the imbalance in the number of cases and controls.

		AUC	SE	SP	CutOff	ACC	TN	TP	FN	FP	NPV	PPV
MiR155_5p		0.797	0.958	0.589	0.03	0.61	1650	158	7	1150	0.996	0.121
**MiR320a**		**0.969**	**0.97**	**0.912**	**0.072**	**0.915**	**2554**	**160**	**5**	**246**	**0.998**	**0.394**
MiR32_5p		0.803	0.982	0.486	0.021	0.514	1362	162	3	1438	0.998	0.101
MiR93_5p		0.694	0.479	0.872	0.095	0.85	2441	79	86	359	0.966	0.18
Combination 1	MiR155_5p-MiR32_5p	0.85	0.879	0.674	0.039	0.685	1886	145	20	914	0.99	0.137
**Combination 2**	**MiR155_5p-MiR320a**	**0.969**	**0.97**	**0.913**	**0.073**	**0.916**	**2557**	**160**	**5**	**243**	**0.998**	**0.397**
Combination 3	MiR155_5p-MiR93_5p	0.81	0.939	0.581	0.03	0.601	1627	155	10	1173	0.994	0.117
**Combination 4**	**MiR32_5p-MiR320a**	**0.969**	**0.976**	**0.905**	**0.058**	**0.909**	**2533**	**161**	**4**	**267**	**0.998**	**0.376**
Combination 5	MiR32_5p-MiR93_5p	0.821	0.867	0.63	0.044	0.644	1765	143	22	1035	0.988	0.121
**Combination 6**	**MiR320a-MiR93_5p**	**0.97**	**0.97**	**0.916**	**0.08**	**0.919**	**2565**	**160**	**5**	**235**	**0.998**	**0.405**
**Combination 7**	**MiR155_5p-MiR32_5p-MiR320a**	**0.969**	**0.976**	**0.908**	**0.061**	**0.912**	**2542**	**161**	**4**	**258**	**0.998**	**0.384**
Combination 8	MiR155_5p-MiR32_5p-MiR93_5p	0.856	0.836	0.73	0.051	0.736	2044	138	27	756	0.987	0.154
**Combination 9**	**MiR155_5p-MiR320a-MiR93_5p**	**0.97**	**0.97**	**0.916**	**0.079**	**0.919**	**2564**	**160**	**5**	**236**	**0.998**	**0.404**
**Combination 10**	**MiR32_5p-MiR320a-MiR93_5p**	**0.969**	**0.976**	**0.913**	**0.07**	**0.916**	**2556**	**161**	**4**	**244**	**0.998**	**0.398**
**Combination 11**	**MiR155_5p-MiR32_5p-MiR320a-MiR93_5p**	**0.97**	**0.976**	**0.913**	**0.071**	**0.917**	**2557**	**161**	**4**	**243**	**0.998**	**0.399**

Supplementary Figure S1. Overview of the diagnostic potential of the best performing miRNA combinations. Ability of the most promising combinations to correctly distinguish pancreatic cancer patients not only from non-cancer controls but also from the other cancer types. Red dotted line indicates optimal cutoff based on ROC analysis in PC cases and non-cancer controls.

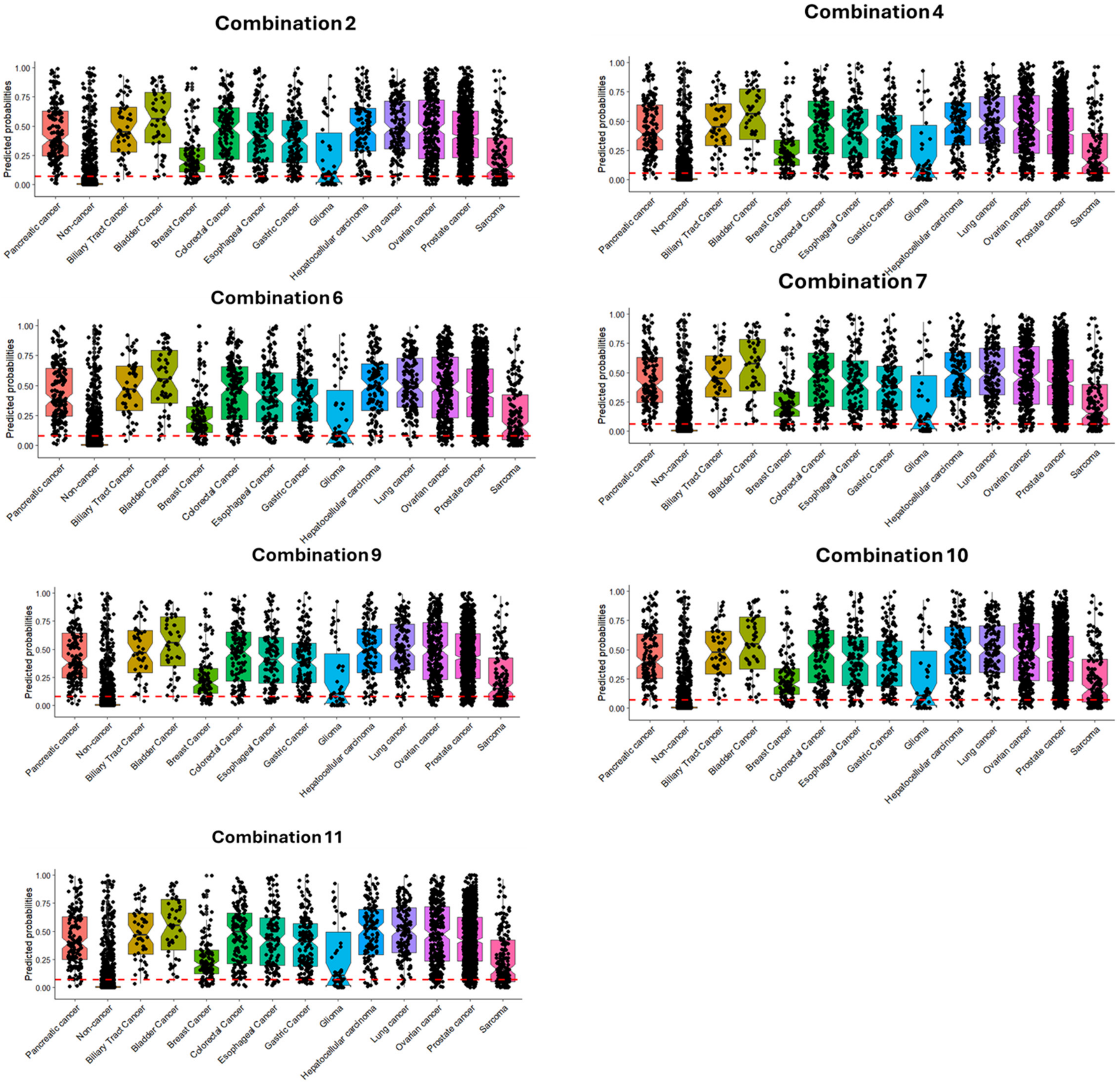

Additional information on SonoWell instrument specifications and experimental reproducibility

The instrument used, SonoWell®, is indeed commercially available from Inno-Sol srl (www.inno-sol.it/SonoWell) since january 2025. The instrument used in this study was a prototype beta-version, and the commercial version was first launched at the International Symposium of Therapeutic Ultrasound (ISTU April, 2023) held in Lyon, France. The same year in June the instrument was certified CE, and tin January 2025, Inno-Sol has started its world-wide commercialization.
The patents protecting the technical solutions to physical problems are well described in the scientific literature and are concerning the ultrasound transducers behavior under stress for long sonication time use, and to those problems arising by the US waves propagation and their interaction with the polystyrene material of the well-plates.
Briefly: 1) Efficiency of transducers conversion of electric pulses into ultrasound waves is affected by over-heating of the transducer's active membrane, this depending on the material nature can induce either a meaningful drop or enhancement of the efficiency, which would translate in a variation of the actual acoustic pressure along the duration of the experiment. The resulting heating would thus affect reproducibility of the acoustic energy emission along the experiment. The SonoWell adopts patented technical solutions that ensures the transducers temperature does not oscillate more than 5°C even (thus the effect is totally neglectable in our experiments) in acquisitions lasting up to three consecutive hours (we didn't test longer times) and even developing much larger acoustic pressures (we tested up to 1.0 MPa). 2) The arrangement of the experiment adopts the "plate-on transducers" format, with the height distance from transducer chosen to position the bottom of the plate at the Near/Far Field distance (-1mm) where the acoustic field is homogeneous along the Z-axis. The patented technical solution adopted within the instrument ensures that this distance is always respected. 3) No cross-contamination of US field crossing through the bottom of the plate to the well nearby to the sonicated well, this is again achieved by another patented technical solution. 4) No overheating of the well plate. It is well known from literature that there is a thermal effect of US impacting on polystyrene material, and this phenomenon needs to be kept under control by a judicious choice of the US parameters.
The instrument SonoWell® is optimized to keep under control all the issues 1-4, providing an excellent reproducibility of measures.
Other technical solutions useful when performing experiments on well-plates, which were not used in the present study since for the acoustic pressures developed and for the design of the experiment they are neglectable, are described within the web site www.inno-sol.it/block-us (technical solution that allows the minimization of the conversion modes of the propagating US wave arising from the wave impact on polystyrene material) and in www.inn-sol.it/inno-cap (technical solution that allows the minimization of the reflection back of the US waves when hitting the interface liquid medium/air).
Other groups may use other type instruments as well, albeit with the caveat that they should successfully reproduce in a stable manner (for all the long sonication duration) the acoustic

parameters we are publishing we expect the results should be reproducible. Of course, on different cell types they should find the best compromise between the successful sonoporation effect and cell viability not compromised (i.e., viability > 85%).
